# The Developing Role of Evidence-Based Environmental Health: Perceptions, Experiences, and Understandings From the Front Line

**DOI:** 10.1177/2158244015611711

**Published:** 2015-10-26

**Authors:** Surindar Dhesi, Jill Stewart

**Affiliations:** 1 University of Manchester, UK; 2 University of Birmingham, UK; 3 University of Greenwich, London, UK

**Keywords:** environmental health, public health, social determinants of health, evidence-based practice, local government

## Abstract

There has been renewed recognition that proactive strategies and interventions can address the social determinants of health, and the environmental health profession is well placed to effect positive change in many of these determinants. This qualitative research has revealed differences in the perceptions, experiences, and understandings of evidence-based practice among public health professionals from different backgrounds across different services in health care and local government in England. The absence of a strong tradition of evidence-based practice in environmental health appears to be a disadvantage in securing funding and playing a full role, as it has become the expectation in the new public health system. This has, at times, resulted in tensions between professionals with different backgrounds and frustration on the part of environmental health practitioners, who have a tradition of responding quickly to new challenges and “getting on with the job.” There is generally a willingness to develop evidence-based practice in environmental health; however, this will take time and investment.

## Introduction

Marmot (2010) states that “most effective actions to reduce health inequalities will come through action within the social determinants of health” (p. 86); however, the public health evidence base and policy decisions are commonly centered on a “downstream” medical concept of evidence-based practice rather than in the “upstream” social determinants of health focused on prevention of ill health (Asthana & Halliday, 2006). This is true even where there is a commitment to tackling “upstream” determinants (Popay, Whitehead, & Hunter, 2010). To illustrate, there has been recent criticism of the Chief Medical Officer’s report on child health focusing on “health-care” services and “individual-level targets” and its failure to suggest action on wider issues (Tillmann, Baker, Crocker-Buque, Rana, & Bouquet, 2014).

Others have also found that while policy commitments to address the social determinants of health are frequently made (in Canada), these are often not implemented; instead, action is focused on promoting individual “healthy lifestyle choices” (Raphael, 2011, p. 222). Just as in England, it is argued that Ontario’s public health units have generally neglected the social determinants of health (Brassolotto, Raphael, & Baldeo, 2014) favoring concentration on individual behavior, which, they argue, depoliticizes the issue.

Environmental health (EH) is rooted in the social determinants, focusing on “upstream” actions, preventing illness, disease, and accident in living and working environments; it

addresses all the physical, chemical, and biological factors external to a person, and all the related factors impacting behaviours. It encompasses the assessment and control of those environmental factors that can potentially affect health. It is targeted towards preventing disease and creating health-supportive environments. (World Health Organization, 2011)

Originally charged to deal with basic public health such as sanitation in the mid-19th century, the role has expanded to include a broad approach to health improvement and protection, including food safety, occupational health and safety, housing, and pollution matters, including contaminated land, smoke, dust, and noise control. EH practitioners are also increasingly involved in tackling other public health issues such as obesity. Rehfuess and Bartram (2014) suggest a useful definition of EH interventions: “any modifications to the natural or physical environment, or behaviours relating directly to them, which are undertaken with the intention to protect or improve public health” (p. 155). They add that such interventions are complex and require methods of evaluation which take into account the many factors involved.

The local authority EH function includes a regulatory or enforcement role, which differentiates it from other public health occupations. There remain tensions in meeting these often narrow regulatory requirements and leading on wider public health, both in policy and practice. Rather than taking an holistic approach, EH has tended to fragment into administrative silos, including food safety, occupational health and safety, housing and environmental protection, and in what has been described as “action-oriented” fields (Eyles, 1997). This, coupled with the challenges of measuring the effectiveness of action on complex public health issues in the short-term (Bauld & Judge, 2008), presents difficulties in developing the evidence base required to persuade decision-makers of the legitimacy and cost-effectiveness of proactive interventions. Consequently, there has been a substantial impact on the ability to adopt an evidence-based system in EH. The development of an evidence base for tackling the social determinants of health, and an evidence base for EH are separate issues. However, they are also highly interlinked and interdependent. We therefore address them together here.

This article presents the findings of empirical research exploring EH practitioners’ perceptions, and the challenges faced, around the adoption and use of evidence-based practice in the new English public health system. Specifically, four key themes are discussed: perceptions of evidence-based practice, practical challenges and their implications, relationships with public health colleagues, and responding to the demand for evidence-based EH.

## Background and Context

Public health arrangements in England recently underwent significant organizational change when health service–based public health practitioners formally moved to local authorities on April 1, 2013. This followed historical restructuring in 1974, when public health medicine joined the National Health Service (Byrne, 1994; Stewart, Bushell, & Habgood, 2003), leaving EH in local authorities, contributing to challenges we describe. Health and Wellbeing Boards (HWBs) went live at the same time as the restructure; these are local government led committees charged with setting the local strategic direction for health, including public health, bringing together representatives from health and local authorities. The new system has required all public health professionals, whether formerly health service or local authority based, to work more closely to improve health and well-being and tackle health inequalities in their local populations (Department of Health, 2012). However, EH practitioners do not have a secure and mandated place on HWBs and have been found to be largely “invisible” to their colleagues in the wider public health system (Dhesi, 2014).

The new arrangements provide an opportunity for investment in “upstream” preventative actions that are evidence based. However, there remain concerns that a medical model which values randomized controlled trials above other forms of evidence will predominate. There is an urgent need for those in EH and their partners to ensure a refocus on the social determinants of health, to tackle the causes and not just the effects of poor health. Importantly, observers have noted that funding for health promotion activities is now linked to evidence-based practice and that “this is now the norm” (Dunne, Scriven, & Furlong, 2012, p. 109). However, they add that the evaluation of work to create this evidence base requires investment, and it is clear that work is required in both policy and practice to establish the effectiveness of preventative strategies and interventions which are the core of EH work.

Looking at the wider context, several commentators have noted the impact of a tough financial climate (D. Hunter, South, & Gamsu, 2014), public service and benefit cuts (Winters, McAteer, & Scott-Samuel, 2012), neoliberal policies (McCartney et al., 2013; Mooney, 2012), and austerity policies particularly affecting deprived areas (Barr & Harrison, 2012), all impacting on public health and widening health inequalities. Indeed EH is located within local government, which has been subject to significant financial cuts in recent years.

## Evidence-Based Public Health

Evidence-based public health is a fairly new idea and has been identified as being “of particular relevance to environmental health” (Rehfuess & Bartram, 2014, p. 155). It has been noted that

because of the complex and often “wicked issues” with which public health wrestles, there is much debate over what counts as evidence and how it can best be applied in different contexts. Evidence doesn’t exist in a vacuum—how it is presented and by whom are key issues which can determine its value and uptake. Even where respected evidence resources exist . . . awareness of them remains poor in many local authorities. And getting their findings into practice locally can be problematic. (D. Hunter et al., 2014)

D. J. Hunter (2009) also adds that “an evidence-informed public health is probably the best that can be hoped for” (p. 586) and Murphy (2013) critically notes the move to policy-based evidence making from evidence-based policy making in recent health reforms.

The concept of public health “evidence” itself is tricky: It can be uncertain, change, and be overruled by politics (Killoran & Kelly, 2004; Stewart, 2005). Others have suggested that science and politics are particularly intertwined in the field of regulatory science (Strassheim & Kettunen, 2014), and Fafard (2015) argues that there should be a reduced focus on scientific evidence in favor of ideas that take into account the realities of how this evidence translates into public health policy.

The evidence base can also be inaccessible to many at the front line of practice; as access by local government public health practitioners to peer-reviewed papers has typically been limited. The evidence relating to the social determinants of health necessarily comprises a range of factors, including information and analysis, surveillance, research, evaluation, local knowledge, and good practice (i.e., what works, and why), and Rehfuess and Bartram (2014) note the value of systematic reviews here.

Marks (2002) identifies “three factors commonly held to have influenced the shift towards evidence based practice in the UK are cost-containment, quality assurance and the purchaser/provider split in the internal market of the NHS” (p. 5), and it may be for this latter reason that a medical model still prevails. Another likely factor is the period of time the medical evidence base has been relied upon and added to, while other evidence bases—for example, in public health, where the situation is often more empirically complex and less easy to tease apart—have lagged behind. As we have described, these areas have developed outside health services and have different and complex functions. In addition, the time lag between interventions and outcomes for public health interventions can be significant, a challenging issue in such a fast moving policy context, where initiatives are often time-limited and subject to change.

Although there have been calls for an evidence base, it is not always clear what this means, what this evidence might look like, and how practitioners might develop their skills and competencies to use and contribute to this evidence base. It has been argued (in nursing) that a wide understanding of what constitutes “evidence” is appropriate, including practitioner and patient experience and local contextual information (Harvey et al., 2004). It is clear that such outcomes can be difficult to measure and require quantitative and qualitative data founded on a range of methods (Asthana & Halliday, 2006) to explain what works well and why. It also needs to be fit for purpose, continually evaluated, and revisable as well as being accessible (Muir Gray, 2000; Trinder, 2000).

## Evidence-Based EH

EH is balanced between regulatory (statutory) functions which are legally required to be carried out and wider non-statutory functions which are discretionary. Elements of the regulatory frameworks in which they operate are not necessarily health outcome specific, for example, performance measurement on the numbers of food hygiene or occupational health and safety inspections carried out. Although hazard and risk have become increasingly factored into regulation, other issues such as a requirement to enhance social capital or community cohesion are not. Prioritizing of regulation in some areas, often as a result of policies of austerity, has left little space for a wider focus in the social determinants of health for those at the front line of EH practice where there is potential for greater health impact. What is lacking is the routine use of, and contribution to, a robust evidence base to shape how EH practitioners tackle the social determinants of health on which their daily work is focused, in ensuring safe living and working conditions for their local populations.

Historically, EH practitioners and other local authority professional groups did have access to service delivery and improvement support, including information on evidence for practice, from organizations such as the Audit Commission, the IDeA, and LACORS; however, Murphy (2014) finds that these forms of support for local authorities have been substantially reduced in recent years, and this is reflected in the findings that we present below.

Of particular relevance here is the suggestion that moving toward a greater focus on the social effectiveness of intervention programmes, based on a “shared understanding between researchers and practitioners,” is needed. This should focus on how social relationships can be reconfigured in public health programmes (Rod, Ingholt, Sørensen, & Tjørnhøj-Thomsen, 2014, p. 9). Interdisciplinary becomes important in practitioner engagement in intervention research, methods, and social theory. This is key in EH, which in many areas tends to revolve around intervention and exit to meet regulatory requirements, and this in itself does not contribute toward a wider social change that could have far greater impact. Crucially, others have found (in town planning) that, where both evidence-based public health guidance exist against regulatory guidance, the former is likely to have limited impact (Allender, Cavill, Parker, & Foster, 2009).

## Method

The results presented here form part of a larger qualitative research project which utilized longitudinal case studies over a period of 18 months, ending in July 2013 to explore how the new English HWBs were tackling health inequalities, focusing on EH. The authors are academics and Environmental Health Practitioners (EHPs) (one actively practicing), and the implications of this on the research, including the use of professional networks, interviewing peers, and dealing with challenging findings, have been reported elsewhere (Dhesi, 2013). All participants in the research were aware of the lead authors’ professional background; the second author was not involved in data collection or analysis. The project was approved by the University of Manchester ethics committee.

Four case study sites in the Midlands and North of England, each a HWB, were followed more than 18 months, from early 2012, and interviews were carried out with EH professionals from all English regions. Multiple case studies were chosen for theoretical replication (Yin, 2015), and case study sites were selected for maximum variation including both unitary and two-tier local government structures, deprived and affluent, and urban and rural areas. The methods used at each case study site were semi-structured interviews with HWB members, support officers, and EH practitioners and managers (50). This was further supported by observation of HWB meetings (20), and analysis of documents produced by HWBs, such as strategies and minutes of meetings.

Each case study site was recruited by approaching the chair of the HWB, either directly following an introduction or through an intermediary. HWB members were asked whether they were willing to take part in the research at the first meeting attended, and all participants were provided with an information sheet or summary of the research. All interviewees gave their informed consent and were given an opportunity at the end of the interview to raise any additional issues they felt were relevant. Interviews were carried out in a location of the interviewee’s choice.

Of the interviews, 24 were with EH practitioners or managers, and their roles are made clear in the findings presented below. EH is a graduate profession, and so levels of education were not explored; however, interviewees were asked to explain their current roles and backgrounds. With the exception of one EH manager who had worked in a related regulatory field, all had worked previously as EH practitioners.

Data was analyzed thematically both inductively and deductively using the qualitative analysis software Atlas ti. and tested for bias with non-EH research colleagues.

## Findings

The findings have been divided into four themes: perceptions of evidence-based practice, practical challenges and their implications, relationships with public health colleagues, and responding to the demand for evidence-based EH. Each is discussed in the context of the literature and with illustrative examples.

### Perceptions of Evidence-Based Practice

A primary challenge was what was understood by evidence-based practice and how this applies to EH. With tensions between regulatory activities and wider public health work, also comes a tension between the social determinants of health and individual lifestyle issues, and action at societal level against a focus on the individual. This relationship between management of existing services and leadership in achieving public health outcomes was important.

There was an expectation that evidence-based practice will be the norm for public health professions in the new system, whatever their backgrounds and employing organizations. EH interviewees, whether practitioners or managers, repeatedly said, “we just get on with it,” lacking the time or resource to take stock to evaluate their actions, and to develop evidence to prioritize for the greatest impact. Indeed, there were no notable differences in opinion between EH practitioners and managers across the thematic findings. EH practitioners follow statutory guidance, codes of practice, and informal evidence based on practical knowledge and experience, but tend not to engage directly with the academic literature.

Established performance indicators were felt to be an issue, and some questioned why food hygiene inspections at specific intervals, for example, received such attention when there was little robust evidence to demonstrate whether this was an effective use of resource (EH practitioner ID45). Interviewees often observed that new approaches are needed to demonstrate their value and secure funding for their services, particularly for discretionary functions, in the new system. This research has also found that EH practitioners see themselves as “doers” compared with other public health colleagues as “thinkers,” and the development and use of evidence to inform practice is key in this perception (EH manager ID1).

Many EH managers felt that relying on fixed outputs rather than public health outcomes as a measurement of effectiveness made them vulnerable to a loss of resources:

. . . if your service is doing well and you don’t have the numbers of prosecutions and notices served, or homelessness cases, you know, there’s a temptation for the [elected] members to think that there’s too much capacity in those areas, they’re thinking there isn’t a problem, therefore, we don’t need so many staff, but it’s actually the front line work that’s going on that’s preventing that kind of thing. (EH manager ID35)

Interestingly, several interviewees reported negative experiences of evidence-based practice and felt that the concept was limiting, both in terms of innovation in dealing with novel problems and in speed of response:

. . . why are we so fixated with “evidence based,” because . . . it actually hampers emerging subjects . . . So it’s like being pioneers of making interventions work—if there’s no evidence there does that mean we can’t have the money to actually do it in the first place? Or is everything a pilot? (EH manager ID1)

Another interviewee expressed concerns that evidence-based practice was being used in a very limited way, resulting in a backward focus:

We only know about what we have been doing, we don’t even research that well enough but we certainly don’t research what we could be doing—and so everybody who is looking at the evidence base is looking for the things they are already doing, well that’s a distortion we can’t live with. (EH practitioner with national role ID33)

As in the wider literature, the view that evidence-based practice was a good thing in itself was clearly not universally held. This seemed to be related to perceptions around limiting the role of professional judgment and room for innovation. This perception is of concern, as evidence-based practice should allow for account to be taken of the context, and of professional judgment. Greenhalgh (2014) in a recent commentary notes that in public health, “success of interventions depends on local feasibility, acceptability and fit with context—and hence on informed, shared decision making with and by local communities . . . ” (p. 3). It is clear that a common understanding of evidence-based practice is needed, which includes room for professional judgment, flexibility, and innovation based on the local context and preferences.

### Practical Challenges and Their Implications

On a practical level, very many interviewees reported feeling unable to provide the evidence required of them and described the challenges of collating credible information, maintaining momentum, and measuring the outcomes and value of EH work. As an example of challenges on the ground, an EH manager (ID40) described frustration when negotiating for time to measure longer-term outcomes in a system where short-term measures are of greater interest, citing a smoking cessation project where funding was threatened when there were no improvements measured after 6 months.

There was a general feeling among interviewees that EH outcomes are difficult to evaluate. This was true across pre-planned inspections around how “worthwhile” they were, as well as wider projects that were frequently not fully evaluated and results disseminated (EH manager ID1). This view is supported by the literature in other fields; for example, social workers face similar issues (Dodd & Epstein, 2012) and others have taken steps to encourage practitioner research and publication and to embed evidence-based practice in social care (Aveyard & Sharp, 2009; Aveyard, Sharp, & Woolliams, 2011; Fronek, 2013). There was seemingly limited consideration of how to develop a body of high quality and persuasive evidence in both policy and practice. Again this sits with a wider literature of how we understand and implement “upstream” evidence-based practice; there is still some way to go.

There also appeared to be a perception that the required evidence will be quantitative or “medical”; however, the concept of what the evidence actually is remained unclear and this was a challenge (EH practitioner ID38). Others felt that there was a lack of the “right sort” of evidence needed to influence decision making:

. . . what I see in terms of what evidence will be used to make decisions and, without a doubt, most of it is medical; there is still a lack of environmental/social evidence, I think that is of higher status and powerful enough to affect decisions, so I think as well, it is much easier to churn out some of the medical data more quickly and some professions have much more of a culture of that than others. (EH practitioner and academic ID42)

There is not simply an issue with a lack of an evidence base; the type of evidence and the way it is presented are seen as crucial in EH being accepted as a public health profession of legitimacy and value and to attract funding as such. For instance, it was said that EH practitioners were carrying out effective work but did not have evidence required to demonstrate their impact to others:

We must be looking at what’s coming up and the innovation that we can do about what’s already here, and getting a research base for that. And the reality is there’s people out there experimenting every day of their life, but they don’t realize they’re doing it, and they’re not recording it, well they’re not doing it in an appropriate way perhaps, but they’re not recording it either, and they’re not sharing, absolutely, except in anecdotes. (EH practitioner with national role ID33)

There was a sense from the research findings that development and use of evidence to inform practice is still in rudimentary stages, and there was little mentioned by way of ideas in what should be done to tackle the “causes of the causes,” and this shortage of “upstream” evidence has also been noted by others (Asthana & Halliday, 2006).

An EH manager considered evidence-based practice a “luxury” rather than a necessity, where resources are tight, but had hopes for future joint working. This perhaps represents the real challenge faced:

We’re a streamlined service, we don’t have much fat on the makeup of the teams and finding time to look into research, look into developing and building baseline data that you can work from is something that we don’t have the luxury of being able to do, that’s one of the things I’m hoping, public health coming in to local authorities might help us with. (EH manager ID31)

It appears that EH needs to urgently respond, if it is to avoid missed opportunities. To illustrate, there were specific concerns that funding would be lost:

. . . there hasn’t been the research done to be able to just go and find a paper that says: “Environmental Health—this project should be funded—because it makes this much impact.” That research doesn’t exist—or it hasn’t been published. (EH practitioner ID45)

Others were concerned that the lack of evidence would affect the ability of EH to engage effectively with HWBs contributing to local public health strategy:

. . . if we really want to have an impact on those Boards and in strategy and also make sure they’ve got the right resource—you have to have the right research and the background to prove your case. (EH manager ID34)

It is clear that interviewees felt that the lack of an evidence base was having an impact not only on the perception of EH as a public health profession but also on the ability to play a full role strategically and in securing funding for services. However, it is also possible that the Joint Strategic Needs Assessment (JSNA) may offer EH practitioners an opportunity to build and develop an evidence base. Others alluded to a skills gap in EH, but this was mentioned infrequently and indirectly, including the fact that much of the information was available but not sufficiently evaluated or disseminated to maximum effect. The loss of Audit Commission National Reports are also significant here, and issues around practical challenges chime with Murphy’s (2014) research findings relating to sports services, where there were concerns around they type of evidence used for decision making, difficulty in evaluating health impacts of interventions, and underdeveloped skills to carry out the evaluation required.

### Relationships With Public Health Colleagues

The perceived need for evidence-based practice has added a layer of complexity, and also sometimes tension between public health professions as they are required to co-operate and may be competing for limited funding. There is optimism, however, that many issues can be overcome by working more closely together, learning from others, and playing to their relative strengths.

Several interviewees reported that the combination of expectation of evidence-based practice and lack of available evidence in EH had caused tensions with public health colleagues from different backgrounds, with one interviewee describing it as “like a religion in medicine” (EH manager ID40). Others described this expectation to follow evidence-based practice as the cause of frustrating delays where fast responses were required, again reinforcing the EH practitioner idea of themselves as “doers,” although their role requires much “thinking”; however, this appears to be unappreciated by many. There was a commonly held view that being “doers,” EH practitioners were at an advantage in able to respond quickly to new and emerging public health issues (EH manager ID46).

One interviewee described an uncomfortable meeting with public health colleagues, when they questioned the use of the medical evidence-based practice norm to secure funding (EH practitioner ID45). There was, however, some hope that the relocation of health service colleagues to local authorities would facilitate a combined skill set able to plug the evidence gap in EH:

. . . if we can make use of analysts, statisticians that are coming in from the PCT [now superseded] we then, possibly, [will] be in a better position to start contributing better and making a stronger argument when it comes to looking at priorities. (EH manager ID31)

Others also reported that colleagues with health service backgrounds had “their finger on the pulse” as regards evidence-based practice and had more success in describing impact. There was hope that the restructure would enable EH (and other services) to learn lessons about accessing and incorporating evidence into practice (EH manager ID4). This squares with wider issues around access to relevant evidence to inform practice; however, an EH manager expressed concerns that the move would lead to a loss of access to the evidence by their former health service colleagues.

Others felt that being able to demonstrate the value of EH work would make an impact in how the profession is perceived:

If we do this and we show the benefits, then it’s going to be a lot of benefit to us, because people will say, “Well look, Environmental Health, they’ve really delivered here.” (EH manager ID36)

The findings indicate that there are tensions between individuals and organizations in the new public health system. Difficulties in partnership working between health and local authorities are not new and have been documented (Evans & Killoran, 2000), with tensions arising from different worldviews, priorities, and ways of working. However, most research has focused on local authority social care; relationships between EH and other health and public health professions are under-researched and this area requires more attention as the new public health system becomes established.

### Responding to the Demand for Evidence-Based EH

To thrive, there is a need to learn a new way for EH to demonstrate impact and effectiveness which will require a completely new approach and a greater sense of equality with public health colleagues. Within the new public health structures, EH managers are starting to consider how they will measure the short- and long-term effects of their work to demonstrate impact and secure funding:

We’ve barely scratched the surface of the analytics of some of the tobacco work, [but] we’ve actually got reasonable numbers about what we’re doing. But big questions about does enforcement influence price? Does it influence availability? What will an elected member get for their money? If they give us another enforcement officer will there be measurable health impact? Are we just a finger in the dam wall and the best we can say is it’s not getting any worse or are we actually making a difference? *If we can actually show a meaningful cause and effect in terms of outcomes for say tobacco work I think the balance of spending from that would be different*. (EH manager ID46; emphasis added)

In response to the practical challenges described earlier, multiple new skills are required, primarily not only in research and evaluation but also in novel forms of dissemination and presentation that attract the attention of those holding the purse strings. As an interviewee described,

. . . it’s how we present ourselves, how we get ourselves on a level playing field really. (EH manager ID1)

A recurrent theme appeared to be a disconnect between “getting on with the job” and reflection around how interventions might be evaluated and enhanced. This area seems particularly lacking, and as Baum (2008) identifies, successful policies and practices need to address underlying causes of inequality and be founded in evidence.

This shift in expectations requires a shift in practice around how new or unused skills in reflection, evaluation, and publication can be factored into already busy working regimes. When asked about why EH did not have a strong tradition of evidence-based practice, a variety of ideas were suggested; however, by far, the most common response was lack of time:

. . . if your job is to . . . crunch out the statistics, because that’s what it comes down to, how do you then find the time and the energy to do the things that actually might be more important and have more of an impact on health? (EH practitioner and academic ID42)

An interviewee, in comparing EH with other public health colleagues, felt that the issues should be overcome:

. . . I spent a couple of days working for the HPA [Health Protection Agency, now superseded], and I noticed how good they are at evidencing what they do—but it’s part of their culture . . . when you read their monthly report book . . . it’s just so professionally done . . . and you think should we be getting more serious about that in Environmental Health? I think we probably should be . . . (EH practitioner ID38)

There were two notable mentions of the successful use of evidence-based practice by EH managers, both working in cities, though in very different geographical areas and circumstances. The first relates to levering in funding for housing interventions to tackle health inequalities through quantifying costs and modeling for savings in health and other spending;

We’ve had very long debates about outcomes and outputs because these things are so difficult to measure, if you’re exposed to substandard housing the symptoms may not manifest themselves for 5, 10, 15 years and there’s no way you can have a sort of impact assessment or evaluation done in a short period of time, but what we are able to do is model . . . So EHOs [environmental health officers] in year 1 cost roughly £300,000 in salaries levered in by in terms of landlord improvements several hundred thousand pounds and will be saving, or are estimated to save the NHS £4.4 million over 10 years and wider society £11 million. (EH manager ID43)

The second manager had used a variety of approaches to demonstrate the effectiveness of their service:

. . . on our project work we’ve done quite a bit of evidence based evaluation but it’s been both qualitative and quantitative, so we have done quite a lot of feedback in a qualitative manner, so interviews as well as the nub of how many referrals, to whom and all that sort of stuff . . . and it is quite difficult when you get asked; right, what are your outcomes, what are you monitoring to actually come up with something that’s useable. Because we tend to deal with things over a longer term so it is quite difficult sometimes, but I think we’re creative. (EH manager ID48)

These examples indicate evidence-based practice is possible within an EH setting and is already being successfully used in some areas to demonstrate the impact of EH work. However, only one of these positive examples has been published, and that in “gray” practitioner publications rather than peer-reviewed literature, and so the opportunity of others in the profession and wider system to learn from these experiences is limited. The value of “gray” literature to practitioners also very much depends on its quality assurance and availability.

There was also some positivity expressed by interviewees around the practical steps that could be taken to start evaluating, including consideration at the planning stage of how success will be measured:

So you’re looking at it at the beginning going; right, okay, well, what do we want to achieve and how are we going to monitor it? Not doing it and then getting halfway through going; what have we done and what have we achieved, you really need to start at the beginning. (EH manager ID48)

An interviewee with a strategic role in a local authority also expressed openness to considering non-medical evidence for public health impacts:

. . . probably it’s easier in housing where there was a bigger national evidence base . . . if . . . the housing intervention costs a few hundred pounds, but could potentially save thousands of pounds in hospital treatment, that’s where you actually do start getting people going, oh yeah, and then you can tie that back to the JSNA [Joint Strategic Needs Assessment] . . . I think it’s about normalizing what you think of as public health. (HWB support officer ID30)

Although much was mentioned around the challenges of evidence-based practice, little was offered by way of concrete action. However, there are some signs that EH practitioners and managers feel that by evaluating their work in terms of outcomes, they will be able to demonstrate the health impacts, and there are indications that some public health decision-makers will be open to this evidence in terms of service funding, including, for discretionary work addressing the social determinants of health. There also needs to be an acknowledgment that time is needed for these activities as part of the “day job” if the best use of limited resources is to be identified.

However, the value of “gray” literature such as technical reports, opinion pieces, and other unpublished work compared with peer-reviewed papers remains unclear in the practical context. Recent housing case studies have been published (Stewart, 2013) and housing “evidence bases” are available; however, it is unclear whether gray literature will be accepted as “evidence” sufficient to influence decision making and funding, and more research is needed.

Rehfuess and Bartram (2014) suggest a new five-stage model for evidence synthesis on EH intervention effectiveness. They take into account the complexity of action on upstream interventions, including the “geographical, socio-economic, political and cultural environment.” The five stages are policy measures, programming, delivery, user compliance, and direct impact. While clearly requiring resources to action, it appears that this could be a useful tool for EH practitioners and managers in the new English public health system to make progress in a systematic way with demonstrating the value of their interventions in terms of public health outcomes.

## Conclusion

Changes in the English public health system have brought the lack of disseminated EH evidence to the fore; in public health medicine, it is already considered relatively well established, and this has caused some tensions. Although in the past there has been some sharing of best practice and other support, those working in local authority EH in particular have in recent years tended not to evaluate their work in terms of health impact and disseminate by publication. This needs to be addressed as a priority, as does continuing measurement on fixed outputs where the public health impacts are unknown.

The very range of EH issues and the fact that some are founded in the social determinants of health and others as fragmented regulatory silos needs to be far more comprehensively addressed in policy and practice, widely understood, and founded in theory of what works and why. The organizational changes present new opportunities for greater multi-disciplinary working and learning, particularly in sharing skills around public health outcomes, access to literature, and opportunities to develop an accessible evidence base.

Many commentators (cited above) argue of the need to ensure a continued focus in the social determinants of health and evidence-based strategies and interventions that will be sustainable. There is a clear need for evidence of impact to be created and published, appropriately disseminated, incorporated into implementation plans, and reflected upon. Rehfuess and Bartram’s (2014) proposed model for systematic evidence synthesis in EH could be a useful tool for practitioners, but this will require investment in expertise, time, and resources.

EH professionals recognize this deficit, and while they may not all agree on the value of evidence-based practice in itself, they see that being able to demonstrate effectiveness in this way is necessary to survive and thrive sufficient to take action on upstream issues, particularly where these actions fall outside the narrow regulatory remit. Interestingly, none of those interviewed referred to the former modes of support for best practice dissemination in local government, which have declined in recent years. There is some optimism among interviewees that the evidence base can be developed, particularly if efforts are made to work with other public health colleagues more familiar with the concept, but in a way that is valid and responds to both theory and practice in EH and health promotion more widely. In this way, EH may gain recognition for its work impacting on the social determinants of health.

The dilemmas faced are multiple. The focus needs to be on establishing evidence-based EH, rooted in actions to tackle the social determinants of health and recognizing the need for flexibility around professional judgment, local context, and preferences; yet required performance indicators frequently skew resources in a different direction. Funding cuts can lead to a retrenchment and focus on statutory functions and less resource to design and evaluate proactive and discretionary wider public health work.

This article has presented data collected during a time of upheaval and change, where relationships and structures were often newly established, and it remains clear that further work is needed when systems are more established. The role of EH as a public health profession is greatly under-researched and is deserving of more attention.
